# Regulation of eukaryotic mRNA deadenylation and degradation by the Ccr4-Not complex

**DOI:** 10.3389/fcell.2023.1153624

**Published:** 2023-04-20

**Authors:** Lorenzo Pavanello, Michael Hall, Gerlof Sebastiaan Winkler

**Affiliations:** ^1^ School of Pharmacy, University of Nottingham, University Park, Nottingham, United Kingdom

**Keywords:** mRNA, deadenylation, degradation, Ccr4-Not, deadenylase, gene regulation, gene expression

## Abstract

Accurate and precise regulation of gene expression programmes in eukaryotes involves the coordinated control of transcription, mRNA stability and translation. In recent years, significant progress has been made about the role of sequence elements in the 3′ untranslated region for the regulation of mRNA degradation, and a model has emerged in which recruitment of the Ccr4-Not complex is the critical step in the regulation of mRNA decay. Recruitment of the Ccr4-Not complex to a target mRNA results in deadenylation mediated by the Caf1 and Ccr4 catalytic subunits of the complex. Following deadenylation, the 5′ cap structure is removed, and the mRNA subjected to 5′-3′ degradation. Here, the role of the human Ccr4-Not complex in cytoplasmic deadenylation of mRNA is reviewed, with a particular focus on mechanisms of its recruitment to mRNA by sequence motifs in the 3′ untranslated region, codon usage, as well as general mechanisms involving the poly(A) tail.

## Introduction

In eukaryotes, accurate and precise regulation of gene expression programmes involves the coordinated control of transcription, mRNA stability and translation. Over the last decade, there has been significant progress in understanding the role of sequence motifs in the 3′ untranslated region on mRNA stability *via* recruitment of proteins involved in 5′-3′ mRNA decay, which is a major pathway for the degradation of cytoplasmic mRNA (Muhlrad et al., 1994; Stoecklin et al., 2006; Bonisch et al., 2007). In particular, the structural basis for understanding critical steps in the pathway have been uncovered. A model has emerged in which mRNA decay is controlled at the level of deadenylation with a key role attributed to the Ccr4-Not deadenylase, which has been shown to interact with a number of mRNA-binding factors. Moreover, deadenylation and subsequent mRNA degradation can also be induced *via* interactions with the poly(A) tail and the ribosome.

In this review, we will focus on mechanisms of recruitment of the Ccr4-Not deadenylase with emphasis on examples where structural information is available and that can serve as paradigms for understanding the regulation of mRNA degradation. Emphasis will be placed on the structure and function of the human proteins with information obtained from the analysis of model organisms included where they provide additional insight. In addition, it should be noted that the Ccr4-Not complex is not only involved in deadenylation, but can also reduce translational efficiency through multiple interactions with proteins involved in translational control (Meijer et al., 2013; Chen et al., 2014; Mathys et al., 2014).

## Deadenylation and the 5′-3′ degradation pathway

**FIGURE 1:**
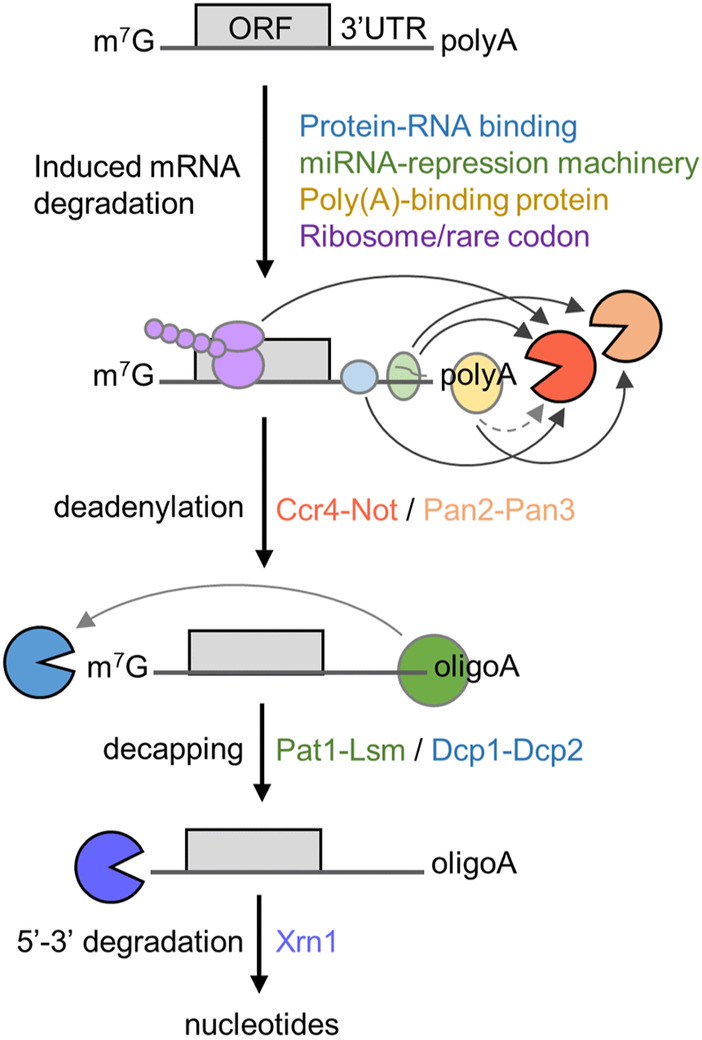
Role of deadenylation in the 5′-3′ degradation pathway. Schematic diagram of the 5′-3′ mRNA degradation pathway. Indicated are RNA proteins regulating mRNA stability, the sequential steps of the 5′-3′ degradation pathway, and protein complexes required. The open reading frame (ORF) and 3′ untranslated region (UTR) are indicated. Arrows indicate direct interactions between components; the dotted line indicates indirect interactions between poly(A)-binding protein and Ccr4-Not.

The 5′-3′ mRNA decay pathway requires the coordinated action and enzymatic activities associated with several multi-subunit protein complexes (Figure 1). The initial and rate-limiting step of this mRNA degradation pathway is the enzymatic shortening of the poly(A) tail (deadenylation) (Parker and Song, 2004; Goldstrohm and Wickens, 2008). There are two main deadenylases involved in this step: the Pan2-Pan3 complex that may complete initial, fast deadenylation, while the Ccr4-Not complex may be engaged in the second, slower phase (Yamashita et al., 2005). Following deadenylation, the Lsm1-7/Pat1 complex binds the 3′ end followed by recruitment of the Dcp1-Dcp2 decapping complex (Chowdhury et al., 2007; Chowdhury et al., 2014). This exposes the 5′ end to exoribonucleolytic attack by the Xrn1 enzyme, which carries out 5′-3′ degradation of the mRNA body (Parker and Song, 2004; Chen and Shyu, 2011; Jonas and Izaurralde, 2015). Notwithstanding the importance of poly(A) tail shortening as the initial step in mRNA decay, the relationship between the length of the poly(A) tail, translational efficiency, and mRNA stability is complex [reviewed in (Passmore and Coller, 2022)]. Indeed, many highly expressed mRNAs that are stable and efficiently translated are characterised by the presence of short poly(A) tails (Peng and Schoenberg, 2005; Lima et al., 2017).

In the 5′-3′ pathway, the main mechanism leading to enhanced deadenylation involves interactions between trans-acting factors binding the mRNA and the Ccr4-Not deadenylase. This includes mRNA-specific mechanisms using sequence elements typically located in the 3′ untranslated region (UTR), that are recognised by trans-acting factors including sequence-specific mRNA binding proteins, and the TNRC6 (GW182) component of the miRNA-repression machinery (Goldstrohm and Wickens, 2008; Jonas and Izaurralde, 2015). However, additional mechanisms of recruitment of the Ccr4-Not deadenylase have also been described. First, binding of the Ccr4-Not complex to the ribosome is linked with the prevalence of low abundance codons and poor translational efficiency in *Saccharomyces cerevisiae* (Buschauer et al., 2020). Secondly, the Ccr4-Not complex can be recruited to the poly(A) tail *via* the cytoplasmic poly(A)-binding protein 1 (PABPC1) (Figure 1) (Ezzeddine et al., 2007; Mauxion et al., 2008; Ezzeddine et al., 2012; Stupfler et al., 2016).

## Structure of the Ccr4-Not deadenylase complex

**FIGURE 2:**
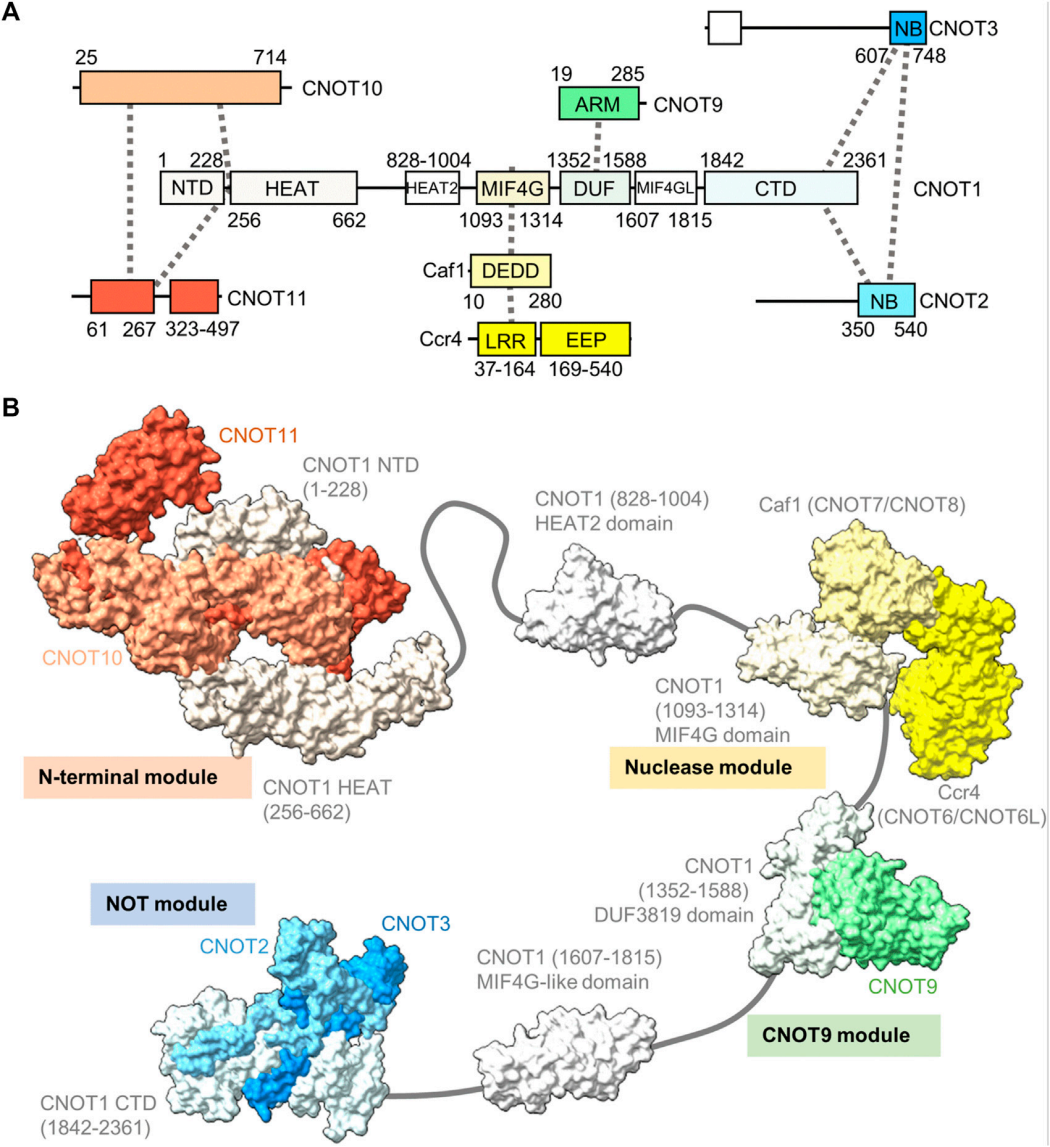
Structure of the Ccr4-Not complex. **(A)** Overview of the architecture of the Ccr4-Not complex. Indicated are the subunits and conserved domains. Interactions are indicated using dotted lines. The colours represent functional modules. **(B)** Structure of the human Ccr4-Not complex. Indicated are the N-terminal module composed of the N-terminal region of CNOT1, CNOT10 (light orange) and CNOT11 (dark orange) (PDB entry: 8BFI) (Mauxion et al., 2022), MIF4G-like domain 1 of CNOT1 (PDB entry:4J8S) (Fabian et al., 2013), the nuclease module composed of the CNOT1 MIF4G domain, Caf1/CNOT7 (light yellow) and Ccr4/CNOT6L (dark yellow) (PDB entries 3NGQ and 7VOI) (Wang et al., 2010; Zhang et al., 2022), the CNOT9 module (PDB entry: 4^−ΔΔCT^6 or 4CRV) (Chen et al., 2014; Mathys et al., 2014) composed of the DUF3819 domain of CNOT1 and CNOT9 (green), a second MIF4G-like domain of CNOT1 (modelled on the structure of the Chaetomium thermophilum fragment, PDB entry 6H3Z) (Raisch et al., 2018), and the NOT module composed of the CNOT1 C-terminal domain and the conserved NOT-Box regions located at the C-termini of CNOT2 (light blue) and CNOT3 (blue) (PDB entry: 4C0D) (Boland et al., 2013). Numbers in brackets refer to the amino acid residues of CNOT1.

The human Ccr4-Not complex has a molecular weight of approximately 675 kDa and contains eight subunits (Figure 2A) (Collart and Panasenko, 2012; Wahle and Winkler, 2013). The complex has a highly conserved core, but there are also some differences between fungal and metazoan complexes in terms of subunit composition (Table 1). Based on single-particle analysis by electron microscopy of yeast Ccr4-Not, the complex has a flat, L-shaped structure (Nasertorabi et al., 2011).

**TABLE 1:** Composition of eukaryotic Ccr4-Not complexes.

*S. cerevisiae*	*S. pombe*	*D. melanogaster*	*H. sapiens*	Comments
Not1	Not1	NOT1	CNOT1	
Not2	Not2	NOT2	CNOT2	
Not3				Homologue of Not5
Not4	Not4	NOT4	CNOT4	Ubiquitin-protein ligase; non-canonical subunit
Not5	Not3	NOT3	CNOT3	Orthologues of Sc Not5
Caf40	Caf40	CAF40	CNOT9	
Caf1	Caf1	CAF1	CNOT7/CNOT8^a^	Ribonuclease (3′-5’; poly(A) selective)
Ccr4	Ccr4	CCR4	CNOT6/CNOT6L^b^	Ribonuclease (3′-5’; poly(A) selective)
		NOT10	CNOT10	
		NOT11	CNOT11	
	Mmi1			Non-canonical subunit
Caf130				Non-canonical subunit

^a^ Caf1 is used when referring to CNOT7 and/or CNOT8.

^b^ Ccr4 is used when referring to CNOT6 and/or CNOT6L.

The backbone of the complex is formed by the large subunit CNOT1 (Not1), which contains at least six structured domains connected by short linkers that display a degree of flexibility (Bawankar et al., 2013). The subunit can be viewed as a string containing beads, with several beads comprising one or two additional subunits forming well-structured, rigid sub-complexes or modules (Figure 2B). Structures for four modules have been determined by x-ray crystallography. Firstly, the N-terminal module is composed of the N-terminal region of CNOT1 that forms a complex interface with CNOT10 and CNOT11 (Mauxion et al., 2022). A relatively long region without known function links the N-terminal module to a MIF4G-like domain, which contains an interaction region with the TTP protein that recognises the AU-rich element in mRNA (Fabian et al., 2013).

The nuclease module is composed of the MIF4G domain and the catalytic subunits Caf1 and Ccr4 (Figure 2B). Caf1 contains an DEDDh nuclease domain that directly interacts with the MIF4G domain of CNOT1 (Not1). In addition, Caf1 interacts with a leucine-rich repeat (LRR) domain at the N-terminus of Ccr4, which is linked to the C-terminal EEP (exonuclease, endonuclease, phosphatase) nuclease domain of Ccr4 (Wang et al., 2010; Basquin et al., 2012; Petit et al., 2012; Chen et al., 2021; Zhang et al., 2022). Both catalytic subunits are encoded by two paralogues in vertebrates: CNOT7 and CNOT8 encode the Caf1 subunit, while CNOT6 and CNOT6L are orthologues of Ccr4. The proteins encoded by the paralogous genes appear to have similar functions, and at the cellular level, both CNOT7 and CNOT8 have largely redundant and overlapping roles in both human and mouse cells (Aslam et al., 2009; Mostafa et al., 2020). Similar observations have been made for CNOT6 and CNOT6L (Mittal et al., 2011; Mostafa et al., 2020). Interestingly, as determined by x-ray crystallography and electron paramagnetic resonance spectroscopy, there is a significant distance (approximately 65 Å in the human complex) between the catalytic centres of Caf1 and Ccr4 indicating that the Caf1 and Ccr4 subunits have unique functions, or that significant conformational changes take place during mRNA deadenylation (Basquin et al., 2012; Zhang et al., 2022).

Caf1 and Ccr4 are both poly(A)-selective ribonucleases (Goldstrohm and Wickens, 2008; Wahle and Winkler, 2013). The presence of guanosine residues inhibits deadenylation, and the inclusion of non-A residues in poly(A) tails by TENT4A (PAPD7) and TENT4B (PAPD5) can prevent rapid deadenylation and mRNA turnover (Lim et al., 2018; Tang and Stowell, 2019; Kim et al., 2020). In the case of Ccr4, selective recognition of poly(A) residues involves specific recognition of adenosine bases by amino acid residues (Wang et al., 2010). By contrast, the Caf1 nuclease forms multiple interactions with the phosphate-sugar backbone without significant base interactions. In this case, recognition of poly(A) is based on the formation of the form A single stranded helical RNA conformation that depends on multiple base-base stacking interactions, which is disrupted by the presence of non-A residues (Tang and Stowell, 2019).

While it is not fully established whether the Caf1 and Ccr4 subunits contribute independently, or act interdependently in reconstituted systems (Maryati et al., 2015; Stowell et al., 2016; Raisch et al., 2019; Chen et al., 2021; Pekovic et al., 2022), their cellular roles are not equivalent. Using knockdown strategies, it has been shown that the Caf1 and Ccr4 paralogues differentially affect deadenylation and gene expression in mammalian cells (Aslam et al., 2009; Mittal et al., 2011; Yi et al., 2018). Moreover, while the Caf1 paralogues are essential for viability of mouse embryonic fibroblasts, cells lacking both Ccr4 paralogues remain viable (Mostafa et al., 2020).

A third module is composed of the DUF3819 domain that interacts with the CNOT9 (CAF40) subunit (Figure 2B) (Chen et al., 2014; Mathys et al., 2014). The DUF3819 domain is connected to the MIF4G domain by a short linker region. The conserved region of CNOT9 (CAF40) protein, the ARM domain, is composed of armadillo repeats and has a crescent-like shape with a positively charged cleft (Garces et al., 2007; Chen et al., 2014; Mathys et al., 2014). Adjacent on the C-terminal end of the CNOT9-interaction domain of CNOT1 (Not1) is a second MIF4G-like domain, which has no known function (Raisch et al., 2018).

The final well-characterised module is the ‘NOT-module’ composed of the C-terminal region of CNOT1 (Not1) that forms a trimeric subcomplex with the NOT-box regions of the CNOT2 (Not2) and CNOT3 (Not5) subunits (Figure 2B) (Bhaskar et al., 2013; Boland et al., 2013). The NOT-Box regions are located in the C-termini of CNOT2 (Not2) and CNOT3 (Not3). Limited structural information is available about the N-terminal extension of CNOT2 (Not2). Similarly, there is limited structural information about the central region of CNOT3 (Not3). By contrast, the N-terminus of CNOT3 (Not3) is highly conserved and forms a three-helix bundle (Buschauer et al., 2020).

Differences between the structure of the yeast and vertebrate complexes are evident in the proteins associating with the N-terminal region of CNOT1 (Not1), which is composed of a large number of α-helical HEAT repeats (Basquin et al., 2012; Mauxion et al., 2022). In fungi, the N-terminus of Not1 provides a platform for the Caf130 subunit, which is not conserved in metazoans (Chen et al., 2001). Instead, in *Drosophila* and human Ccr4-Not, the N-terminal region of CNOT1 provides an interaction surface for binding to the CNOT10 and CNOT11 subunits (Bawankar et al., 2013; Mauxion et al., 2013; Mauxion et al., 2022). In *Schizosaccharomyces pombe*, the YTH-domain protein Mmi1 is a stable component of the complex (Ukleja et al., 2016). The Mmi1 protein directs removal of meiotic mRNAs containing DSR sequence elements in the 3’ UTR during vegetative growth (Harigaya et al., 2006). In a reconstituted system, Mmi1 recognises the DSR element, and stimulates deadenylation of substrates containing the DSR sequence (Stowell et al., 2016). In *S. pombe*, Mmi1 is also required for the formation of heterochromatin at meiotic genes and sub-telomeric DNA in a manner that is dependent on the nuclease subunits (Cotobal et al., 2015).

Another difference between fungal and metazoan complexes is the association of the CNOT4 (Not4) subunit. This protein provides ubiquitin-protein ligase activity by binding the E2 ubiquitin-conjugating enzyme UbcH5b (Ubc4/5 in yeast) (Dominguez et al., 2004; Bhaskar et al., 2015). In fungi, the Not4 subunit is stably associated with the large subunit Not1 (Bhaskar et al., 2015). By contrast, the protein is not stably attached to metazoan complexes, where a short peptide motif in the C-terminus of CNOT4 (NOT4) interacts with CNOT9 (NOT9) in addition to the C-terminal domain of CNOT1 (Keskeny et al., 2019).

## Regulation of mRNA deadenylation by sequence elements in 3’ UTR

There are several mechanisms for the recruitment of the Ccr4-Not complex that are controlled by sequence elements -typically located in the 3’ UTR of the target mRNA. First, sequence elements can be recognised by protein-RNA interactions in a manner analogous to the recognition of DNA promoter elements by transcription factors. Sequence elements recognised by protein-RNA interactions are not necessarily linear sequences, but can include RNA structural elements, or covalent base modifications. Alternatively, Ccr4-Not recruitment by the miRNA-repression machinery involves recognition of the sequence element by RNA-RNA base pairing.

### Recruitment of the Ccr4-Not deadenylase by RNA-binding proteins

The notion that recruitment of the Ccr4-Not deadenylase can occur *via* direct interactions with RNA-binding regulators came from observations from diverse experimental systems. For example, in *S. cerevisiae*, it was shown that Mpt5p, a member of the Pumilio family of proteins that are known regulators of mRNA stability and translation, binds directly to the Caf1 subunit, and induces mRNA deadenylation by Ccr4 (Goldstrohm et al., 2006; Goldstrohm et al., 2007). The interaction between Pumilio proteins and Ccr4-Not is conserved in humans (Goldstrohm et al., 2006). In *Drosophila*, Ccr4-Not was shown to play an important role in development, for example, *via* interactions with the RNA-binding proteins Smaug and Bicaudal-C (Zaessinger et al., 2006; Chicoine et al., 2007). In human cells, the A/U-rich element (ARE)-binding protein Tristetraprolin TTP (ZFP36) was also shown to interact directly with subunits of the Ccr4-Not complex (Lykke-Andersen and Wagner, 2005; Sandler et al., 2011). Interestingly, *C*th2, the yeast orthologue of ZFP36, mediates ARE-mediated decay and is also able to interact with the Ccr4-Not complex (Perea-Garcia et al., 2020).

Using knockdown or knockout strategies, Ccr4-Not is also implicated in the destabilisation of specific transcripts in mammalian cells. For instance, using a liver-specific conditional knockout Cnot1 mouse model, it was demonstrated that Ccr4-Not targets mRNAs for degradation through interactions with ZFP36L1 (butyrate response factor 1), which recognises ARE elements in the 3’ UTR of mRNAs, and Ago2, a component of the miRNA-repression complex (Takahashi et al., 2020). By contrast, in 4T1 breast cancer cells, CNOT7 preferentially regulates levels of mRNAs containing the cytoplasmic polyadenylation element (CPE), Pumilio binding element (PUM), Nanos response elements (NRE), and cleavage and polyadenylation stimulation factor binding element (CPSF) (Faraji et al., 2016). Interestingly, in this model, transcripts containing the ARE were not preferentially targeted by Ccr4-Not (Faraji et al., 2016).

Below, we discuss several interactions between RNA-binding regulators and the Ccr4-Not complex for which structural information is available. Interestingly, in many cases, interactions are mediated by relatively short peptide motifs present in the RNA-binding protein, and structured domains of Ccr4-Not subunits.

### Roquin

**FIGURE 3:**
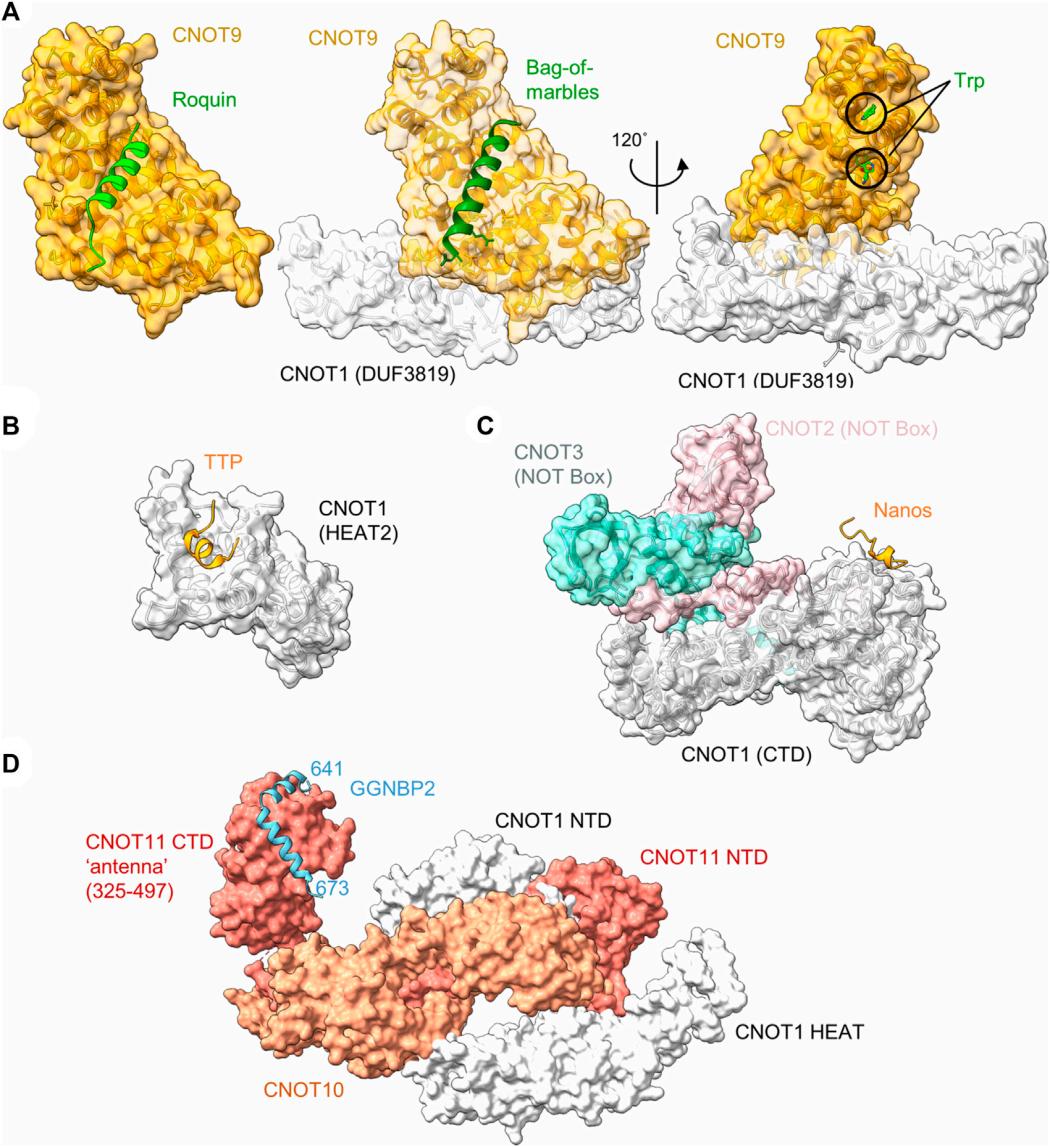
Recruitment of Ccr4-Not by regulators of mRNA stability. **(A)** Interaction of peptide motifs recognised by the CNOT9 (CAF40) subunit. *Left panel*, *Drosophila* CAF40 in complex with a peptide of the RNA-binding protein Roquin. PDB entry: 5LSW (Sgromo et al., 2017). *Middle panel*, *Drosophila* NOT1-CAF40 in complex with a peptide from the RNA binding protein Bag-of-Marbles. PDB entry: 5ONA (Sgromo et al., 2018). *Right panel*, Human CNOT1-CNOT9 in complex with two tryptophan residues. PDB entry: 4CRV (Chen et al., 2014) or 4^−ΔΔCT^7 (Mathys et al., 2014). **(B)** Interaction of a peptide from the RNA-binding protein Nanos in complex with C-terminal domain (CTD) of human CNOT1 (PDB entry: 4CQO) (Bhandari et al., 2014). The model includes the position of the NOT-Box regions of CNOT2 and CNOT3 obtained by superposition of PDB entry 4C0D (Boland et al., 2013). **(C)** Binding of a TTP peptide to a MIF4G-like domain of CNOT1. PDB entry: 4J8S (Fabian et al., 2013). **(D)** Model of the N-terminal module in complex with a fragment of GGNBP2. The model was created by superimposing PDB entries 8BFI and 8BFJ (Mauxion et al., 2022).

Tumour necrosis factor α (TNF-α) is a pro-inflammatory cytokine, which is expressed in multiple cell types, including monocytes and macrophages. Tight control of the expression levels of TNF-α must be maintained, which involve multiple sequence elements in the 3’ UTR of the TNF-α mRNA, including an A/U-rich element (ARE), and a constitutive decay element (CDE) which is 37 nucleotides in length (Tan et al., 2014). In the active conformation, the CDE forms an RNA stem loop structure which can be recognised by the Roquin proteins, which in turn recruit the Ccr4-Not complex to the mRNA (Leppek et al., 2013). The N-terminal ROQ domain facilitates binding of Roquin 1 to the CDE RNA element (Tan et al., 2014), while the C-terminal region is able to associate with CNOT1 (Leppek et al., 2013). Even though the C-terminal region of Roquin 1 is not conserved, human Roquin 2 and *Drosophila* Roquin also interact with Ccr4-Not *via* their C-terminal regions *via* multiple short motifs (Sgromo et al., 2017). One interaction motif was mapped to around 23 amino acids, which can form an amphipathic α-helical structure. This peptide motif binds directly to the concave side of CNOT9 (CAF40) *via* multiple hydrophobic interactions (Figure 3A) (Sgromo et al., 2017).

### Bag-of-marbles (Bam)

Bag-of-marbles (Bam) is a determinant of the fate of germ cells in *Drosophila*. It is conserved in diptera and has no orthologues in human and mouse cells (Sgromo et al., 2018). It is not clear whether Bam binds RNA directly, or whether it influences RNA recognition of other proteins, such as Pumilio (Malik et al., 2019). Regardless, Bam can directly induce mRNA degradation and repress translation *via* interactions with Ccr4-Not. This ability is confined to an N-terminal α-helical region of 24 amino acids, which folds into an amphipathic helix that interacts with the CNOT9 (CAF40) subunit of Ccr4-Not (Sgromo et al., 2018). The interaction is mediated *via* hydrophobic residues that bind the concave side of CNOT9 and overlaps with the binding site of Roquin (Figure 3A).

Interestingly, the Bam/Roquin binding site of CNOT9 is also exploited by proteins that are not directly involved in recognition of RNA sequence elements. The CNOT4 (NOT4) subunit is not a core subunit in *Drosophila* and human Ccr4-Not complexes. Again, this subunit interacts with sites on multiple Ccr4-Not subunits, including CNOT1 and CNOT9. In case of CNOT9, a 23-amino acid motif that is conserved between *Drosophila* and human mediates the interaction of CNOT4 (NOT4) (Keskeny et al., 2019). The binding site of this motif overlaps with those of Bam and Roquin. In addition, the E3 ubiquitin protein-ligase RNF219 also binds the same interface of CNOT9 *via* a short peptide motif (Poetz et al., 2021).

### Tristetraprolin

Tristetraprolin (TTP), also known as zinc finger protein (ZFP) 36, is a small RNA-binding protein, which is implicated in the regulation of components of the inflammatory response. TTP is well conserved, with members of the TTP family found in all major groups of the eukaryotes (Patial and Blackshear, 2016). TTP targets for degradation those mRNAs which contain AU-rich elements in the 3′UTR. The interaction between TTP and the AU-rich element is conferred by a central tandem zinc finger (TZF) domain (Patial and Blackshear, 2016). TTP recruits the Ccr4-Not complex *via* multiple short interaction motifs present in both the N- and C-termini of TTP, which can interact with the CNOT1 and CNOT9 subunits (Sandler et al., 2011; Fabian et al., 2013; Bulbrook et al., 2018). Motifs interacting with the CNOT9 subunit contain multiple tryptophan residues that are recognised by W-binding motifs of CNOT9 (Figure 3A) (Bulbrook et al., 2018) and are also involved in miRNA-mediated repression (see below) (Chen et al., 2014; Mathys et al., 2014). The W-binding pockets do not overlap with the concave area of CNOT9 involved in recognition of the amphipathic a-helical interaction motifs of Roquin and Bam (Figure 3A).

In addition to tryptophan-containing interaction motifs, TTP also binds to CNOT1 *via* a short peptide region located at the C-terminus of TTP. The domain of CNOT1 (amino acids 828-1,004) interacting with the TTP peptide forms four helix-turn-helix motifs that are similar to the MIF4G domain that is part of the nuclease module and interacts with the Caf1/CNOT7 subunit (Fabian et al., 2013) (Figure 3B). The interaction of the TTP peptide with the N-terminal part of the CNOT1 domain is mediated *via* central hydrophobic interactions, as well as electrostatic interactions (Fabian et al., 2013).

### Nanos

In most vertebrates, three paralogues of the Nanos protein exist. For example, in the mouse, Nanos 1 is expressed in oocytes and the adult brain, Nanos 2 in male primordial germ cells (PGCs) and Nanos 3 in PGCs of both sexes (Bhandari et al., 2014). The Nanos proteins contain a conserved CCHC-type zinc finger domain which facilitates its binding with the 3′UTR of target mRNAs. Vertebrate Nanos 1 has a short, 17-amino acid NOT1-interacting motif containing an FxxWxDYxxL consensus sequence. This motif directly interacts with the C-terminal region of CNOT1 (Figure 3C) (Bhandari et al., 2014). Nanos proteins do not contain this sequence in all organisms surveyed. For example, the motif is absent in some invertebrate organisms, including *Drosophila*. In this organism, Nanos interacts with the Ccr4-Not complex using redundant motifs that interact with the NOT module (Raisch et al., 2016). While the Ccr4-Not interacting peptides of *Drosophila* Nanos are longer (approx. 30-60 amino acids) than the short peptide motifs found in vertebrate Nanos, one of the motifs also interacts with the NOT-module of the Ccr4-Not complex. Interestingly, the Ccr4-Not-interaction peptide from *Drosophila* Nanos requires the intact NOT-module for interaction, and is bound on a different interaction surface compared to the short interaction motif present in vertebrate Nanos (Raisch et al., 2016).

Another RNA-binding protein interacting with the C-terminal region of CNOT1 is YTHDF2 (Du et al., 2016). This protein selectively recognises a sequence element containing an adenosine methylated at the *N* ^6^ position (G^**m**^ **A**CU/A) *via* its C-terminal YTH domain (Li et al., 2014). This abundant RNA base modification is conserved in eukaryotes, and is involved in a number of processes important for the regulation of gene expression including pre-mRNA processing, nuclear export, mRNA degradation, and translation (He and He, 2021). Recruitment of YTHDF2 to a reporter mRNA containing fragments from the PLAC2 lncRNA that are known to be modified by adenosine methylation, or artificial tethering to a reporter mRNA results in enhanced deadenylation and mRNA degradation (Du et al., 2016). YTHDF2 is highly similar to YTHDF1 and YTHDF3 (overall 55.9% identity, 77.9% similarity). The region of YTHDF2 interacting with CNOT1 was mapped to amino acids 101–200. This region is also highly conserved in YTHDF1 and YTHDF3 (50% identity, 86% similarity), suggesting that YTHDF1 and YTHDF3 may also interact with CNOT1. While no structural details are currently available, the structural information currently available suggests that the NOT-module is a platform for interactions with multiple regulators of mRNA stability.

### Gametogenetin binding protein 2 (GGNBP2)

Gametogenetin binding protein 2 (GGNBP2) is a ubiquitously expressed gene involved in spermatogenesis and the regulation of cancer cell proliferation and metastasis. While there is relatively little information about the function of the gene, the protein binds to the N-terminal module of the Ccr4-Not complex *via* CNOT11 (Figure 3D). In this case, the interaction region of GGNBP2 comprises approximately 32 amino acids, which form two α-helices that wrap around the HEAT repeats of the C-terminal region of CNOT11 (Mauxion et al., 2022). Because knockdown of CNOT10 in mouse ES cells results in upregulation of a large number of mRNAs (Du et al., 2020), it is tempting to speculate that recruitment of Ccr4-Not *via* the N-terminal module can serve as a mechanism for the regulation of mRNA levels (Mauxion et al., 2022).

## Recruitment of Ccr4-not by the miRNA-repression machinery

Repression mediated by miRNAs is conserved in metazoan organisms. Recognition of the regulatory sequence in the mRNA is mediated by imperfect base pairing of the miRNA. The impact on translation and mRNA deadenylation and decay is mediated *via* the RNA-induced silencing complex (RISC), which contains several proteins including the AGO proteins that bind the single stranded miRNA, and the glycine-tryptophan (GW) rich protein TNRC6 (GW182). Tethering TNRC6 (GW182) to a reporter mRNA mimics repression induced by a miRNA-target site indicating that TNRC6 (GW182) is a critical component of the RNA-induced silencing complex (Pillai et al., 2004).

The TNRC6 (GW182) protein can bind to the Ccr4-Not complex *via* interactions with the CNOT1 and CNOT9 subunits. Regarding CNOT1, TNRC6 (GW182) directly interacts with CNOT1*via* two LWG-repeat motifs in the C-terminal domain that both bind to CNOT1 (Braun et al., 2011; Chekulaeva et al., 2011; Fabian et al., 2011). In addition, TNRC6 (GW182) can interact with the CNOT9 subunit of Ccr4-Not (Chen et al., 2014; Mathys et al., 2014). In this case, two binding pockets for tryptophan residues have been identified on the convex surface of CNOT9 (Figure 3A) (Chen et al., 2014; Mathys et al., 2014). As mentioned above, these pockets were also found to be important for the interaction with peptide motifs of TTP (Bulbrook et al., 2018) and do not overlap with the area on the concave side of CNOT9 that interacts with amphipathic helical peptide motifs from Bam or Roquin (Sgromo et al., 2017; Sgromo et al., 2018).

## Recruitment of the Ccr4-Not deadenylase to mRNAs containing rare codons

**FIGURE 4:**
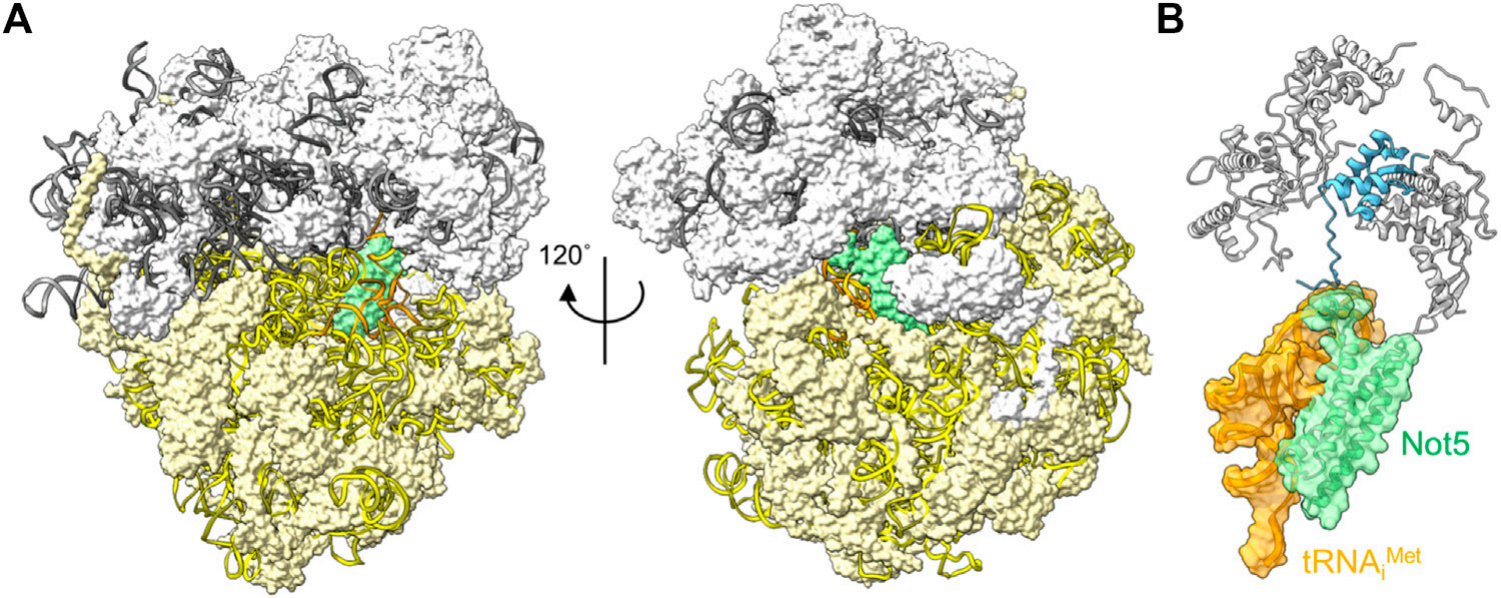
Interactions between the ribosome and Ccr4-Not subunits. **(A)** Complex of the *S. cerevisiae* ribosome with an empty A-site, the N-terminal domain of Not5 located in the E-site (green), and tRNA_i_^Met^ occupying the P-site (orange). The small 40S (white) and large 60S (light yellow) ribosome subunits are indicated; 18S rRNA (yellow); 5S, 5.8S and 25S rRNA (grey) are represented using ribbons. **(B)** Detail of the interactions of *S. cerevisiae* Not5 located in the E-site (green) with the tRNA_i_^Met^ occupying the P-site (orange) and the small ribosome subunit S25 (blue). PDB entry 6TB3 (Buschauer et al., 2020).

The correlation between codon usage, transcript stability and translational efficiency has been observed in eukaryotes ranging from yeast to human cells (Hoekema et al., 1987; Presnyak et al., 2015; Bazzini et al., 2016; Wu et al., 2019). Transcripts containing open-reading frames containing the most abundant codons (major codons) are more efficiently translated, and display greater stability compared to transcripts containing less abundant codons (minor codons) that are less efficiently translated. The mechanistic connection between these processes has been elusive, but recent experiments carried out in the budding yeast *S. cerevisiae* offer a possible mechanism. Work by Buschauer et al. showed that the N-terminal domain of yeast Not5, the orthologue of human CNOT3, can bind ribosomes in the E-site when the A-site is empty (Buschauer et al., 2020) (Figure 4A). The E-site is available for binding by Not5 when minor codons are encountered and the A-site is unoccupied due to the low abundance of the incoming tRNA-aminoacyl complex. The N-terminal region of Not5 in the E-site contains three helices and makes extensive interactions with the tRNA in the P-site (Figure 4B). Both ribosomes containing the initiator tRNA (tRNA_i_^Met^) in the P-site, as well as elongating ribosomes containing the nascent polypeptide can be bound by CNOT3 (Buschauer et al., 2020). It can be envisaged that recruitment of the Ccr4-Not complex *via* Not5 results in deadenylation and subsequent decay by the 5′-3′ degradation pathway, providing a mechanism linking the presence of minor codons, translational efficiency and mRNA stability. Interestingly, binding of Not5 is dependent on ubiquitylation of the ribosomal eS7 subunit by Not4 and requires tRNA to be present in the P-site (Panasenko and Collart, 2012; Ikeuchi et al., 2019; Buschauer et al., 2020; Allen et al., 2021).

Binding of the N-terminal domain of CNOT3 into the ribosomal E-site is conserved in mammalian cells (Absmeier et al., 2022). Intriguingly, mutations in CNOT3 have been associated with T-cell acute lymphoblastic leukaemia (ALL) (De Keersmaecker et al., 2013). A recurring mutation in CNOT3 involves the substitution of a conserved amino acid (Arg-57 corresponding to Lys-58 in yeast) in the N-terminal domain of CNOT3. The equivalent amino acid is presumed to make an important contribution in the interaction between Not5 and the ribosome in yeast (Buschauer et al., 2020). *De novo* and inherited mutations in CNOT3 are also associated with the intellectual developmental disorder with speech delay, autism, and dysmorphic facies (IDDSADF) suggesting that the recruitment of Ccr4-Not by CNOT3 to transcripts with minor codons and low translational efficiency is essential for development (Meyer et al., 2020).

## Poly(A)-mediated recruitment of deadenylation

A third mechanism by which Ccr4-Not can be recruited to mRNA involves members of the BTG/TOB family of proteins and the cytoplasmic poly(A)-binding protein 1 (PABPC1). This mechanism appears to be unique to vertebrates, as no homologues of BTG/TOB proteins have been identified in fungi or *Drosophila* (Mauxion et al., 2009; Winkler, 2010).

**FIGURE 5:**
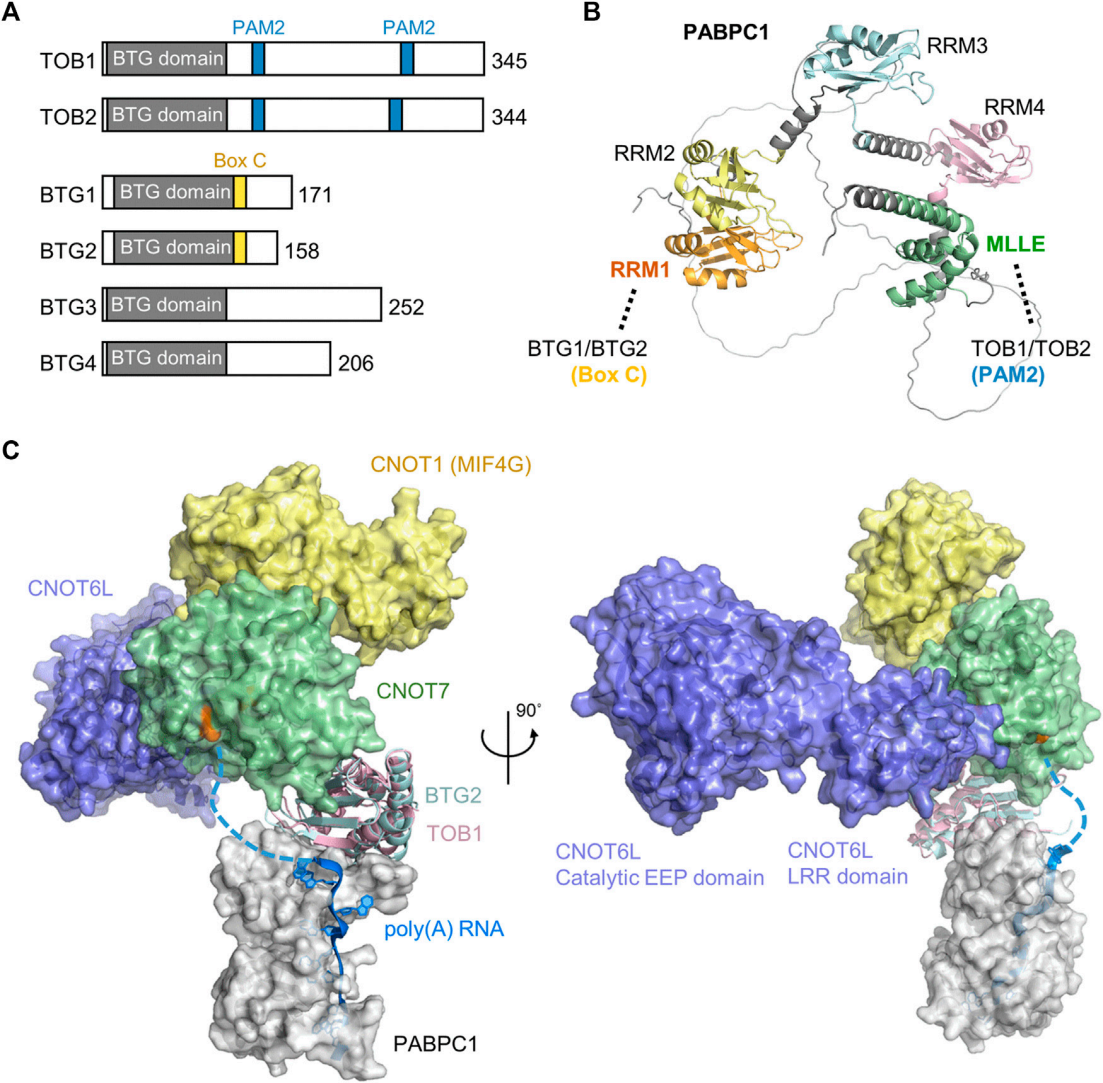
Recruitment of the Ccr4-Not complex by PABPC1. **(A)** Overview of the BTG/TOB proteins. Indicated are the conserved BTG (APRO) domain (grey), PAM2 motifs present in TOB1 and TOB2 (blue), and Box C regions in BTG1 and BTG2 (yellow). **(B)** Model of Hs PABPC1 (Uniprot P11940) (Jumper et al., 2021). Indicated are RRM1 (orange) that interacts with Box C regions of BTG1/BTG2, RRM2-RRM4 (yellow, light blue, pink), and the MLLE domain (green) interacting with PAM2 motifs of TOB1/TOB2. **(C)** Model of the human nuclease module in complex with BTG2-PABPC1 (RRM1-2)-poly(A) (PDB_Dev entry PDBDEV_00000099) (Ameerul et al., 2022). Indicated are PABPC1 (white); BTG2 (light blue); TOB1 (plum); Caf1/CNOT7 (light green); Ccr4/CNOT6L (purple); CNOT1 (MIF4G domain, yellow) and poly (A) RNA (blue). Highlighted (orange, black arrow) are active site residues of Caf1/CNOT7 (Asp-40, Glu-42, Asp-161, His-225, and Asp-230).

The BTG/TOB proteins are characterised by an N-terminal domain that directly interacts with the Caf1 catalytic subunits of Ccr4-Not (Yang et al., 2008; Horiuchi et al., 2009) (Figure 5A). In addition to the conserved N-terminal domain, family members contain divergent C-terminal extensions. Six BTG/TOB proteins are encoded in the human genome: the paralogues TOB1 and TOB2, the paralogues BTG1 and BTG2, and the more distantly related proteins BTG3 and BTG4. These proteins share the ability to repress proliferation and cell-cycle progression upon overexpression, which depends on the interaction with the Caf1 subunit of Ccr4-Not (Ezzeddine et al., 2007; Doidge et al., 2012; Ezzeddine et al., 2012; Stupfler et al., 2016).

While BTG1/BTG2 and TOB1/TOB2 directly interact with PABPC1 and stimulate deadenylation by Ccr4-Not in the presence of PABPC1, different mechanisms of recruitment are involved. TOB1/TOB2 contain the well-characterised PAM2 motif (PABP-interacting Motif 2) in their C-termini. This short peptide motif (approximately 12 amino acids) binds to the C-terminal MLLE domain (PABC) of PABPC1 (Figures 5A,B) (Xie et al., 2014). This domain (approximately 70 amino acid residues) is named after a four amino acid motif, MLLE, that is central to recognition of the PAM2 motif. Although TOB1/TOB2 variants that are unable to interact with PABPC1 fail to stimulate mRNA deadenylation and degradation, artificial recruitment of the proteins to mRNA can induce mRNA deadenylation and degradation (Ezzeddine et al., 2007; Ezzeddine et al., 2012). Similarly, TOB1/TOB2 variants unable to interact with the Caf1 subunit of the Ccr4-Not complex do not induce mRNA deadenylation and degradation (Ezzeddine et al., 2007; Ezzeddine et al., 2012). This suggests a model in which the TOB1/TOB2 proteins are recruited to target mRNAs by interactions between the PAM2 motifs of TOB1/TOB2 and the MLLE domain of PABPC1 resulting in mRNA deadenylation and degradation by recruitment of the Ccr4-Not complex. Interestingly, phosphorylation of unstructured regions surrounding the PAM2 motifs by c-Jun amino-terminal kinase (JNK) can reduce interactions between the TOB2 and PABPC1 suggesting a possible regulatory mechanism that controls deadenylation by Ccr4-Not by TOB1/TOB2 (Huang et al., 2013). By contrast, phosphorylation of a specific residue within the PAM2 motif (Ser-254 of TOB2) occurs *via* a JNK-independent pathway and enhances the interaction between PABPC1 and TOB2 (Chen et al., 2020). In addition to recruitment *via* PABPC1, TOB1 -but not BTG1/BTG2-can be targeted to mRNAs by the cytoplasmic polyadenylation element-binding proteins (CPEBs) (Hosoda et al., 2011; Ogami et al., 2014; Poetz et al., 2022). In this case, a short peptide motif located between the BTG domain and the first PAM2 motif is required (Hosoda et al., 2011).

More recently, BTG1/BTG2 were also shown to interact with PABPC1 (Stupfler et al., 2016). However, in this case, the interaction is mediated by a short motif, Box C region of BTG1/BTG2. This motif, comprised of approximately 11 amino acids, is located adjacent to the conserved BTG domain of BTG1/BTG2, and mediates interactions with the first N-terminal RNA Recognition Motif (RRM1) of PABPC1 (Stupfler et al., 2016; Amine et al., 2021; Ameerul et al., 2022). As is the case for TOB1/TOB2, a BTG2 variant that is unable to interact with PABPC1 does not inhibit cell cycle progression indicating that both the ability to bind Ccr4-Not and PABPC1 are important for the function of BTG2 (Stupfler et al., 2016). A recently published model of a quaternary Caf1-BTG2-PABPC1-RNA complex suggests how deadenylation can be stimulated by BTG2 in the presence of PABPC1 (Figure 5C) (Ameerul et al., 2022). In the model, the 3′ end of poly(A) bound to PABPC1 is directly oriented towards the active site residues of Caf1, which degrades the poly(A) tail in a 3′-5′ direction.

Stimulation of deadenylation by the Caf1 subunit by PABPC1 (Stupfler et al., 2016; Ameerul et al., 2022) appears to be contradictory to a model in which PABPC1 inhibits Caf1 activity and the Ccr4 subunit is active on poly(A) tails containing poly(A)-binding protein (Webster et al., 2018; Yi et al., 2018). A possible explanation could be that there is no orthologue of BTG1/BTG2 in *S. pombe*. In addition, BTG1/BTG2 appear to be downregulated in many mammalian cell lines. Alternatively, PABPC1 recruits the Caf1 subunit *via* BTG1/BTG2 when bound at a short distance from the 3’ end. In this scenario, Caf1 deadenylates the free, unbound terminal end of the poly(A) tail, while its recruitment is stimulated by PABCP1 and BTG1/BTG2.

Deadenylation initiated by interactions involving BTG/TOB proteins, Ccr4-Not and PABPC1 suggests a global mechanism of poly(A) control. Indeed (over-)expression of TOB2 induces a global change in poly(A) tail length and mRNA levels in U2OS cells ectopically expressing TOB2 (Chen et al., 2020). A role for BTG1 and BTG2 in the control of global poly(A)-tail length has also been proposed. Both proteins are important for the maintenance of T-cell quiescence, a state of low proliferation and cellular metabolism. In the absence of both BTG1 and BTG2, proliferation is increased, and spontaneous T-cell activation occurs. Moreover, in native T-cells lacking BTG1 and BTG2, global mRNA levels are elevated, and the global poly(A)-tail length increased (Hwang et al., 2020). Interestingly, recurrent mutations in BTG1 and BTG2 are found in diffuse, large B-cell lymphoma (DLBCL) (Morin et al., 2011; Lohr et al., 2012). This may suggest that global regulation of poly(A) tails may be dysregulated in this type of non-Hodgkin lymphoma, and contributes to malignant transformation.

## Concluding remarks

In recent years, a model has emerged in which mRNA degradation is regulated at the step of recruitment of the Ccr4-Not deadenylase. Recruitment of the deadenylase can be achieved in different ways. Specific mRNAs can be regulated by interactions mediated by proteins recognising sequence elements in the target mRNA, including elements recognised by miRNAs or protein-RNA interactions. These interactions typically involve sequences located in the 3’ UTR. Several examples illustrate how recruitment of the Ccr4-Not deadenylase is achieved. Roquin and Bag-of-marbles contain short peptide motifs that can bind to the concave surface of the CNOT9 (CAF40) subunit. TTP and the TNRC6 (GW182) also bind CNOT9, but in this case interact with the W-binding motifs on the convex side of CNOT9 (CAF40). In addition, TTP and TNRC6 use additional, distinct interaction surfaces of CNOT1. In the case of Nanos, peptide motifs bind the C-terminal region of CNOT1.

A second mode of Ccr4-Not recruitment provides a mechanistic link between codon usage and mRNA stability. The discovery that the N-terminal domain of *S. cerevisiae* Not5 can directly interact with the ribosome when the A-site for the incoming aminoacyl⋅tRNA is unoccupied has provided a molecular mechanism explaining a well-established correlation between codon usage, translational efficiency and mRNA stability. Finally, it has become clear that general mechanisms of Ccr4-Not recruitment involving interactions between PABPC1, members of the BTG/TOB protein family and the Caf1 subunit of Ccr4-Not can lead to global regulation of poly(A) tail length in vertebrates. Exactly how this process can contribute to accurate regulation of gene expression programmes relating to cell proliferation and differentiation remains unclear, even though the importance is illustrated by T-cell activation and cell transformation. In each of these cases, structural information is available for key interactions. Thus, while questions remain, a structural view of regulated deadenylation and mRNA degradation is emerging.

## Data Availability

The original contributions presented in the study are included in the article, further inquiries can be directed to the corresponding author.

## References

[B1] Absmeier E. Chandrasekaran V. O’reilly F. J. Stowell J. Rappsilber J. Passmore L. A. (2022). Specific recognition and ubiquitination of slow-moving ribosomes by human CCR4-NOT. BioRxiv 2022, 501325. 10.1101/2022.07.24.501325 PMC7615087 37653243

[B2] Allen G. E. Panasenko O. O. Villanyi Z. Zagatti M. Weiss B. Pagliazzo L. (2021). Not4 and Not5 modulate translation elongation by Rps7A ubiquitination, Rli1 moonlighting, and condensates that exclude eIF5A. Cell Rep. 36, 109633. 10.1016/j.celrep.2021.109633 34469733

[B3] Ameerul A. Almasmoum H. Pavanello L. Dominguez C. Winkler G. S. (2022). Structural model of the human BTG2-PABPC1 complex by combining mutagenesis, NMR chemical shift perturbation data and molecular docking. J. Mol. Biol. 434, 167662. 10.1016/j.jmb.2022.167662 35640718

[B4] Amine H. Ripin N. Sharma S. Stoecklin G. Allain F. H. Séraphin B. (2021). A conserved motif in human BTG1 and BTG2 proteins mediates interaction with the poly(A) binding protein PABPC1 to stimulate mRNA deadenylation. RNA Biol. 18, 2450–2465. 10.1080/15476286.2021.1925476 34060423 PMC8632095

[B5] Aslam A. Mittal S. Koch F. Andrau J. C. Winkler G. S. (2009). The ccr4-not deadenylase subunits CNOT7 and CNOT8 have overlapping roles and modulate cell proliferation. Mol. Biol. Cell 20, 3840–3850. 10.1091/mbc.e09-02-0146 19605561 PMC2735483

[B6] Basquin J. Roudko V. V. Rode M. Basquin C. Seraphin B. Conti E. (2012). Architecture of the nuclease module of the yeast ccr4-not complex: The not1-caf1-ccr4 interaction. Mol. Cell 48, 207–218. 10.1016/j.molcel.2012.08.014 22959269

[B7] Bawankar P. Loh B. Wohlbold L. Schmidt S. Izaurralde E. (2013). NOT10 and C2orf29/NOT11 form a conserved module of the CCR4-NOT complex that docks onto the NOT1 N-terminal domain. RNA Biol. 10, 228–244. 10.4161/rna.23018 23303381 PMC3594282

[B8] Bazzini A. A. Del Viso F. Moreno-Mateos M. A. Johnstone T. G. Vejnar C. E. Qin Y. (2016). Codon identity regulates mRNA stability and translation efficiency during the maternal-to-zygotic transition. EMBO J. 35, 2087–2103. 10.15252/embj.201694699 27436874 PMC5048347

[B9] Bhandari D. Raisch T. Weichenrieder O. Jonas S. Izaurralde E. (2014). Structural basis for the Nanos-mediated recruitment of the CCR4-NOT complex and translational repression. Genes Dev. 28, 888–901. 10.1101/gad.237289.113 24736845 PMC4003280

[B10] Bhaskar V. Basquin J. Conti E. (2015). Architecture of the ubiquitylation module of the yeast Ccr4-Not complex. Structure 23, 921–928. 10.1016/j.str.2015.03.011 25914052 PMC4431670

[B11] Bhaskar V. Roudko V. Basquin J. Sharma K. Urlaub H. Seraphin B. (2013). Structure and RNA-binding properties of the Not1-Not2-Not5 module of the yeast Ccr4-Not complex. Nat. Struct. Mol. Biol. 20, 1281–1288. 10.1038/nsmb.2686 24121231

[B12] Boland A. Chen Y. Raisch T. Jonas S. Kuzuoglu-Ozturk D. Wohlbold L. (2013). Structure and assembly of the NOT module of the human CCR4-NOT complex. Nat. Struct. Mol. Biol. 20, 1289–1297. 10.1038/nsmb.2681 24121232

[B13] Bonisch C. Temme C. Moritz B. Wahle E. (2007). Degradation of hsp70 and other mRNAs in Drosophila via the 5' 3' pathway and its regulation by heat shock. J. Biol. Chem. 282, 21818–21828. 10.1074/jbc.M702998200 17545151

[B14] Braun J. E. Huntzinger E. Fauser M. Izaurralde E. (2011). GW182 proteins directly recruit cytoplasmic deadenylase complexes to miRNA targets. Mol. Cell 44, 120–133. 10.1016/j.molcel.2011.09.007 21981923

[B15] Bulbrook D. Brazier H. Mahajan P. Kliszczak M. Fedorov O. Marchese F. P. (2018). Tryptophan-mediated interactions between tristetraprolin and the CNOT9 subunit are required for CCR4-NOT deadenylase complex recruitment. J. Mol. Biol. 430, 722–736. 10.1016/j.jmb.2017.12.018 29291391

[B16] Buschauer R. Matsuo Y. Sugiyama T. Chen Y. H. Alhusaini N. Sweet T. (2020). The Ccr4-Not complex monitors the translating ribosome for codon optimality. Science 368, eaay6912. 10.1126/science.aay6912 368 32299921 PMC8663607

[B17] Chekulaeva M. Mathys H. Zipprich J. T. Attig J. Colic M. Parker R. (2011). miRNA repression involves GW182-mediated recruitment of CCR4-NOT through conserved W-containing motifs. Nat. Struct. Mol. Biol. 18, 1218–1226. 10.1038/nsmb.2166 21984184 PMC3885283

[B18] Chen C. A. Strouz K. Huang K. L. Shyu A. B. (2020). Tob2 phosphorylation regulates global mRNA turnover to reshape transcriptome and impact cell proliferation. RNA 26, 1143–1159. 10.1261/rna.073528.119 32404348 PMC7430666

[B19] Chen C. Y. Shyu A. B. (2011). Mechanisms of deadenylation-dependent decay. Wiley Interdiscip. Rev. RNA 2, 167–183. 10.1002/wrna.40 21957004 PMC3624885

[B20] Chen J. Rappsilber J. Chiang Y. C. Russell P. Mann M. Denis C. L. (2001). Purification and characterization of the 1.0 MDa CCR4-NOT complex identifies two novel components of the complex. J. Mol. Biol. 314, 683–694. 10.1006/jmbi.2001.5162 11733989

[B21] Chen Y. Boland A. Kuzuoglu-Ozturk D. Bawankar P. Loh B. Chang C. T. (2014). A DDX6-CNOT1 complex and W-binding pockets in CNOT9 reveal direct links between miRNA target recognition and silencing. Mol. Cell 54, 737–750. 10.1016/j.molcel.2014.03.034 24768540

[B22] Chen Y. Khazina E. Izaurralde E. Weichenrieder O. (2021). Crystal structure and functional properties of the human CCR4-CAF1 deadenylase complex. Nucleic Acids Res. 49, 6489–6510. 10.1093/nar/gkab414 34038562 PMC8216464

[B23] Chicoine J. Benoit P. Gamberi C. Paliouras M. Simonelig M. Lasko P. (2007). Bicaudal-C recruits CCR4-NOT deadenylase to target mRNAs and regulates oogenesis, cytoskeletal organization, and its own expression. Dev. Cell 13, 691–704. 10.1016/j.devcel.2007.10.002 17981137

[B24] Chowdhury A. Kalurupalle S. Tharun S. (2014). Pat1 contributes to the RNA binding activity of the Lsm1-7-Pat1 complex. RNA 20, 1465–1475. 10.1261/rna.045252.114 25035297 PMC4138329

[B25] Chowdhury A. Mukhopadhyay J. Tharun S. (2007). The decapping activator Lsm1p-7p-Pat1p complex has the intrinsic ability to distinguish between oligoadenylated and polyadenylated RNAs. RNA 13, 998–1016. 10.1261/rna.502507 17513695 PMC1894922

[B26] Collart M. A. Panasenko O. O. (2012). The Ccr4-not complex. Gene 492, 42–53. 10.1016/j.gene.2011.09.033 22027279

[B27] Cotobal C. Rodriguez-Lopez M. Duncan C. Hasan A. Yamashita A. Yamamoto M. (2015). Role of Ccr4-Not complex in heterochromatin formation at meiotic genes and subtelomeres in fission yeast. Epigenetics Chromatin 8, 28. 10.1186/s13072-015-0018-4 26279681 PMC4536793

[B28] De Keersmaecker K. Atak Z. K. Li N. Vicente C. Patchett S. Girardi T. (2013). Exome sequencing identifies mutation in CNOT3 and ribosomal genes RPL5 and RPL10 in T-cell acute lymphoblastic leukemia. Nat. Genet. 45, 186–190. 10.1038/ng.2508 23263491 PMC5547913

[B29] Doidge R. Mittal S. Aslam A. Winkler G. S. (2012). The anti-proliferative activity of BTG/TOB proteins is mediated via the Caf1a (CNOT7) and Caf1b (CNOT8) deadenylase subunits of the Ccr4-not complex. PLoS One 7, e51331. 10.1371/journal.pone.0051331 23236473 PMC3517456

[B30] Dominguez C. Bonvin A. M. Winkler G. S. Schaik Van Timmers H. T. Boelens R. (2004). Structural model of the UbcH5B/CNOT4 complex revealed by combining NMR, mutagenesis, and docking approaches. Structure 12, 633–644. 10.1016/j.str.2004.03.004 15062086

[B31] Du H. Chen C. Wang Y. Yang Y. Che Z. Liu X. (2020). RNF219 interacts with CCR4-NOT in regulating stem cell differentiation. J. Mol. Cell Biol. 12, 894–905. 10.1093/jmcb/mjaa061 33104214 PMC7883825

[B32] Du H. Zhao Y. He J. Zhang Y. Xi H. Liu M. (2016). YTHDF2 destabilizes m(6)A-containing RNA through direct recruitment of the CCR4-NOT deadenylase complex. Nat. Commun. 7, 12626. 10.1038/ncomms12626 27558897 PMC5007331

[B33] Ezzeddine N. Chang T. C. Zhu W. Yamashita A. Chen C. Y. Zhong Z. (2007). Human TOB, an antiproliferative transcription factor, is a poly(A)-binding protein-dependent positive regulator of cytoplasmic mRNA deadenylation. Mol. Cell Biol. 27, 7791–7801. 10.1128/MCB.01254-07 17785442 PMC2169145

[B34] Ezzeddine N. Chen C. Y. Shyu A. B. (2012). Evidence providing new insights into TOB-promoted deadenylation and supporting a link between TOB's deadenylation-enhancing and antiproliferative activities. Mol. Cell Biol. 32, 1089–1098. 10.1128/MCB.06370-11 22252318 PMC3295015

[B35] Fabian M. R. Cieplak M. K. Frank F. Morita M. Green J. Srikumar T. (2011). miRNA-mediated deadenylation is orchestrated by GW182 through two conserved motifs that interact with CCR4-NOT. Nat. Struct. Mol. Biol. 18, 1211–1217. 10.1038/nsmb.2149 21984185

[B36] Fabian M. R. Frank F. Rouya C. Siddiqui N. Lai W. S. Karetnikov A. (2013). Structural basis for the recruitment of the human CCR4-NOT deadenylase complex by tristetraprolin. Nat. Struct. Mol. Biol. 20, 735–739. 10.1038/nsmb.2572 23644599 PMC4811204

[B37] Faraji F. Hu Y. Yang H. H. Lee M. P. Winkler G. S. Hafner M. (2016). Post-transcriptional control of tumor cell autonomous metastatic potential by CCR4-NOT deadenylase CNOT7. PLoS Genet. 12, e1005820. 10.1371/journal.pgen.1005820 26807845 PMC4726497

[B38] Garces R. G. Gillon W. Pai E. F. (2007). Atomic model of human Rcd-1 reveals an armadillo-like-repeat protein with *in vitro* nucleic acid binding properties. *Protein science* . a Publ. Protein Soc. 16, 176–188. 10.1110/ps.062600507 PMC2203284 17189474

[B39] Goldstrohm A. C. Wickens M. (2008). Multifunctional deadenylase complexes diversify mRNA control. Nat. Rev. Mol. Cell Biol. 9, 337–344. 10.1038/nrm2370 18334997

[B40] Goldstrohm A. C. Hook B. A. Seay D. J. Wickens M. (2006). PUF proteins bind Pop2p to regulate messenger RNAs. Nat. Struct. Mol. Biol. 13, 533–539. 10.1038/nsmb1100 16715093

[B41] Goldstrohm A. C. Seay D. J. Hook B. A. Wickens M. (2007). PUF protein-mediated deadenylation is catalyzed by Ccr4p. J. Biol. Chem. 282, 109–114. 10.1074/jbc.M609413200 17090538

[B42] Harigaya Y. Tanaka H. Yamanaka S. Tanaka K. Watanabe Y. Tsutsumi C. (2006). Selective elimination of messenger RNA prevents an incidence of untimely meiosis. Nature 442, 45–50. 10.1038/nature04881 16823445

[B43] He P. C. He C. (2021). m(6) A RNA methylation: from mechanisms to therapeutic potential. EMBO J. 40, e105977. 10.15252/embj.2020105977 33470439 PMC7849164

[B44] Hoekema A. Kastelein R. A. Vasser M. Boer De (1987). Codon replacement in the PGK1 gene of *Saccharomyces cerevisiae*: Experimental approach to study the role of biased codon usage in gene expression. Mol. Cell Biol. 7, 2914–2924. 10.1128/mcb.7.8.2914-2924.1987 2823108 PMC367910

[B45] Horiuchi M. Takeuchi K. Noda N. Muroya N. Suzuki T. Nakamura T. (2009). Structural basis for the antiproliferative activity of the Tob-hCaf1 complex. J. Biol. Chem. 284, 13244–13255. 10.1074/jbc.M809250200 19276069 PMC2676056

[B46] Hosoda N. Funakoshi Y. Hirasawa M. Yamagishi R. Asano Y. Miyagawa R. (2011). Anti-proliferative protein Tob negatively regulates CPEB3 target by recruiting Caf1 deadenylase. EMBO J. 30, 1311–1323. 10.1038/emboj.2011.37 21336257 PMC3094127

[B47] Huang K. L. Chadee A. B. Chen C. Y. Zhang Y. Shyu A. B. (2013). Phosphorylation at intrinsically disordered regions of PAM2 motif-containing proteins modulates their interactions with PABPC1 and influences mRNA fate. RNA 19, 295–305. 10.1261/rna.037317.112 23340509 PMC3677241

[B48] Hwang S. S. Lim J. Yu Z. Kong P. Sefik E. Xu H. (2020). mRNA destabilization by BTG1 and BTG2 maintains T cell quiescence. Science 367, 1255–1260. 10.1126/science.aax0194 32165587

[B49] Ikeuchi K. Tesina P. Matsuo Y. Sugiyama T. Cheng J. Saeki Y. (2019). Collided ribosomes form a unique structural interface to induce Hel2-driven quality control pathways. EMBO J. 38, e100276. 10.15252/embj.2018100276 38 30609991 PMC6396155

[B50] Jonas S. Izaurralde E. (2015). Towards a molecular understanding of microRNA-mediated gene silencing. Nat. Rev. Genet. 16, 421–433. 10.1038/nrg3965 26077373

[B51] Jumper J. Evans R. Pritzel A. Green T. Figurnov M. Ronneberger O. (2021). Highly accurate protein structure prediction with AlphaFold. Nature 596, 583–589. 10.1038/s41586-021-03819-2 34265844 PMC8371605

[B52] Keskeny C. Raisch T. Sgromo A. Igreja C. Bhandari D. Weichenrieder O. (2019). A conserved CAF40-binding motif in metazoan NOT4 mediates association with the CCR4-NOT complex. Genes Dev. 33, 236–252. 10.1101/gad.320952.118 30692204 PMC6362812

[B53] Kim D. Lee Y. S. Jung S. J. Yeo J. Seo J. J. Lee Y. Y. (2020). Viral hijacking of the TENT4-ZCCHC14 complex protects viral RNAs via mixed tailing. Nat. Struct. Mol. Biol. 27, 581–588. 10.1038/s41594-020-0427-3 32451488

[B54] Leppek K. Schott J. Reitter S. Poetz F. Hammond M. C. Stoecklin G. (2013). Roquin promotes constitutive mRNA decay via a conserved class of stem-loop recognition motifs. Cell 153, 869–881. 10.1016/j.cell.2013.04.016 23663784

[B55] Li F. Zhao D. Wu J. Shi Y. (2014). Structure of the YTH domain of human YTHDF2 in complex with an m(6)A mononucleotide reveals an aromatic cage for m(6)A recognition. Cell Res. 24, 1490–1492. 10.1038/cr.2014.153 25412658 PMC4260351

[B56] Lim J. Kim D. Lee Y. S. Ha M. Lee M. Yeo J. (2018). Mixed tailing by TENT4A and TENT4B shields mRNA from rapid deadenylation. Science 361, 701–704. 10.1126/science.aam5794 30026317

[B57] Lima S. A. Chipman L. B. Nicholson A. L. Chen Y. H. Yee B. A. Yeo G. W. (2017). Short poly(A) tails are a conserved feature of highly expressed genes. Nat. Struct. Mol. Biol. 24, 1057–1063. 10.1038/nsmb.3499 29106412 PMC5877826

[B58] Lohr J. G. Stojanov P. Lawrence M. S. Auclair D. Chapuy B. Sougnez C. (2012). Discovery and prioritization of somatic mutations in diffuse large B-cell lymphoma (DLBCL) by whole-exome sequencing. Proc. Natl. Acad. Sci. U. S. A. 109, 3879–3884. 10.1073/pnas.1121343109 22343534 PMC3309757

[B59] Lykke-Andersen J. Wagner E. (2005). Recruitment and activation of mRNA decay enzymes by two ARE-mediated decay activation domains in the proteins TTP and BRF-1. Genes Dev. 19, 351–361. 10.1101/gad.1282305 15687258 PMC546513

[B60] Malik S. Jang W. Park S. Y. Kim J. Y. Kwon K. S. Kim C. (2019). The target specificity of the RNA binding protein Pumilio is determined by distinct co-factors. Biosci. Rep. 39. 10.1042/BSR20190099 39 PMC6549094 31097674

[B61] Maryati M. Airhihen B. Winkler G. S. (2015). The enzyme activities of Caf1 and Ccr4 are both required for deadenylation by the human Ccr4-Not nuclease module. Biochem. J. 469, 169–176. 10.1042/BJ20150304 25944446 PMC4613498

[B62] Mathys H. Basquin J. Ozgur S. Czarnocki-Cieciura M. Bonneau F. Aartse A. (2014). Structural and biochemical insights to the role of the CCR4-NOT complex and DDX6 ATPase in microRNA repression. Mol. Cell 54, 751–765. 10.1016/j.molcel.2014.03.036 24768538

[B63] Mauxion F. Basquin J. Ozgur S. Rame M. Albrecht J. Schafer I. (2022). The human CNOT1-CNOT10-CNOT11 complex forms a structural platform for protein-protein interactions. Cell Rep. 42 (1), 111902. 10.1016/j.celrep.2022.111902 36586408 PMC9902336

[B64] Mauxion F. Chen C. Y. Seraphin B. Shyu A. B. (2009). BTG/TOB factors impact deadenylases. Trends Biochem. Sci. 34, 640–647. 10.1016/j.tibs.2009.07.008 19828319 PMC2787745

[B65] Mauxion F. Faux C. Seraphin B. (2008). The BTG2 protein is a general activator of mRNA deadenylation. Embo J. 27, 1039–1048. 10.1038/emboj.2008.43 18337750 PMC2323266

[B66] Mauxion F. Preve B. Seraphin B. (2013). C2ORF29/CNOT11 and CNOT10 form a new module of the CCR4-NOT complex. RNA Biol. 10, 267–276. 10.4161/rna.23065 23232451 PMC3594285

[B67] Meijer H. A. Kong Y. W. Lu W. T. Wilczynska A. Spriggs R. V. Robinson S. W. (2013). Translational repression and eIF4A2 activity are critical for microRNA-mediated gene regulation. Science 340, 82–85. 10.1126/science.1231197 23559250

[B68] Meyer R. Begemann M. Demuth S. Kraft F. Dey D. Schuler H. (2020). Inherited cases of CNOT3-associated intellectual developmental disorder with speech delay, autism, and dysmorphic facies. Clin. Genet. 98, 408–412. 10.1111/cge.13819 32720325

[B69] Mittal S. Aslam A. Medica R. Winkler G. S. (2011). The Ccr4a (CNOT6) and Ccr4b (CNOT6L) deadenylase subunits of the human Ccr4-Not complex contribute to the prevention of cell death and senescence. Mol. Biol. Cell 22, 748–758. 10.1091/mbc.E10-11-0898 21233283 PMC3057700

[B70] Morin R. D. Mendez-Lago M. Mungall A. J. Goya R. Mungall K. L. Corbett R. D. (2011). Frequent mutation of histone-modifying genes in non-Hodgkin lymphoma. Nature 476, 298–303. 10.1038/nature10351 21796119 PMC3210554

[B71] Mostafa D. Takahashi A. Yanagiya A. Yamaguchi T. Abe T. Kureha T. (2020). Essential functions of the CNOT7/8 catalytic subunits of the CCR4-NOT complex in mRNA regulation and cell viability. RNA Biol. 17, 403–416. 10.1080/15476286.2019.1709747 31924127 PMC6999631

[B72] Muhlrad D. Decker C. J. Parker R. (1994). Deadenylation of the unstable mRNA encoded by the yeast MFA2 gene leads to decapping followed by 5'-->3' digestion of the transcript. Genes Dev. 8, 855–866. 10.1101/gad.8.7.855 7926773

[B73] Nasertorabi F. Batisse C. Diepholz M. Suck D. Bottcher B. (2011). Insights into the structure of the CCR4-NOT complex by electron microscopy. FEBS Lett. 585, 2182–2186. 10.1016/j.febslet.2011.05.071 21669201 PMC3171648

[B74] Ogami K. Hosoda N. Funakoshi Y. Hoshino S. (2014). Antiproliferative protein Tob directly regulates c-myc proto-oncogene expression through cytoplasmic polyadenylation element-binding protein CPEB. Oncogene 33, 55–64. 10.1038/onc.2012.548 23178487

[B75] Panasenko O. O. Collart M. A. (2012). Presence of Not5 and ubiquitinated Rps7A in polysome fractions depends upon the Not4 E3 ligase. Mol. Microbiol. 83, 640–653. 10.1111/j.1365-2958.2011.07957.x 22243599

[B76] Parker R. Song H. (2004). The enzymes and control of eukaryotic mRNA turnover. Nat. Struct. Mol. Biol. 11, 121–127. 10.1038/nsmb724 14749774

[B77] Passmore L. A. Coller J. (2022). Roles of mRNA poly(A) tails in regulation of eukaryotic gene expression. Nat. Rev. Mol. Cell Biol. 23, 93–106. 10.1038/s41580-021-00417-y 34594027 PMC7614307

[B78] Patial S. Blackshear P. J. (2016). Tristetraprolin as a therapeutic target in inflammatory disease. Trends Pharmacol. Sci. 37, 811–821. 10.1016/j.tips.2016.07.002 27503556 PMC5030171

[B79] Pekovic F. Rammelt C. Kubíková J. Metz J. Jeske M. Wahle E. (2022). RNA binding proteins Smaug and Cup induce CCR4-NOT-dependent deadenylation of the nanos mRNA in a reconstituted system. BioRxiv 2022, 491288. 10.1101/2022.05.11.491288 PMC10164591 36951092

[B80] Peng J. Schoenberg D. R. (2005). mRNA with a <20-nt poly(A) tail imparted by the poly(A)-limiting element is translated as efficiently *in vivo* as long poly(A) mRNA. RNA 11, 1131–1140. 10.1261/rna.2470905 15929942 PMC1237109

[B81] Perea-Garcia A. Miro P. Jimenez-Lorenzo R. Martinez-Pastor M. T. Puig S. (2020). Sequential recruitment of the mRNA decay machinery to the iron-regulated protein Cth2 in *Saccharomyces cerevisiae* . Biochim. Biophys. Acta Gene Regul. Mech. 1863, 194595. 10.1016/j.bbagrm.2020.194595 32565401

[B82] Petit A. P. Wohlbold L. Bawankar P. Huntzinger E. Schmidt S. Izaurralde E. (2012). The structural basis for the interaction between the CAF1 nuclease and the NOT1 scaffold of the human CCR4-NOT deadenylase complex. Nucleic Acids Res. 40, 11058–11072. 10.1093/nar/gks883 22977175 PMC3510486

[B83] Pillai R. S. Artus C. G. Filipowicz W. (2004). Tethering of human Ago proteins to mRNA mimics the miRNA-mediated repression of protein synthesis. Rna 10, 1518–1525. 10.1261/rna.7131604 15337849 PMC1370638

[B84] Poetz F. Corbo J. Levdansky Y. Spiegelhalter A. Lindner D. Magg V. (2021). RNF219 attenuates global mRNA decay through inhibition of CCR4-NOT complex-mediated deadenylation. Nat. Commun. 12, 7175. 10.1038/s41467-021-27471-6 34887419 PMC8660800

[B85] Poetz F. Lebedeva S. Schott J. Lindner D. Ohler U. Stoecklin G. (2022). Control of immediate early gene expression by CPEB4-repressor complex-mediated mRNA degradation. Genome Biol. 23, 193. 10.1186/s13059-022-02760-5 36096941 PMC9465963

[B86] Presnyak V. Alhusaini N. Chen Y. H. Martin S. Morris N. Kline N. (2015). Codon optimality is a major determinant of mRNA stability. Cell 160, 1111–1124. 10.1016/j.cell.2015.02.029 25768907 PMC4359748

[B87] Raisch T. Bhandari D. Sabath K. Helms S. Valkov E. Weichenrieder O. (2016). Distinct modes of recruitment of the CCR4-NOT complex by Drosophila and vertebrate Nanos. EMBO J. 35, 974–990. 10.15252/embj.201593634 26968986 PMC5207322

[B88] Raisch T. Chang C. T. Levdansky Y. Muthukumar S. Raunser S. Valkov E. (2019). Reconstitution of recombinant human CCR4-NOT reveals molecular insights into regulated deadenylation. Nat. Commun. 10, 3173. 10.1038/s41467-019-11094-z 31320642 PMC6639331

[B89] Raisch T. Sandmeir F. Weichenrieder O. Valkov E. Izaurralde E. (2018). Structural and biochemical analysis of a NOT1 MIF4G-like domain of the CCR4-NOT complex. J. Struct. Biol. 204, 388–395. 10.1016/j.jsb.2018.10.009 30367941

[B90] Sandler H. Kreth J. Timmers H. T. Stoecklin G. (2011). Not1 mediates recruitment of the deadenylase Caf1 to mRNAs targeted for degradation by tristetraprolin. Nucleic Acids Res. 39, 4373–4386. 10.1093/nar/gkr011 21278420 PMC3105394

[B91] Sgromo A. Raisch T. Backhaus C. Keskeny C. Alva V. Weichenrieder O. (2018). Drosophila Bag-of-marbles directly interacts with the CAF40 subunit of the CCR4-NOT complex to elicit repression of mRNA targets. RNA 24, 381–395. 10.1261/rna.064584.117 29255063 PMC5824357

[B92] Sgromo A. Raisch T. Bawankar P. Bhandari D. Chen Y. Kuzuoglu-Ozturk D. (2017). A CAF40-binding motif facilitates recruitment of the CCR4-NOT complex to mRNAs targeted by Drosophila Roquin. Nat. Commun. 8, 14307. 10.1038/ncomms14307 28165457 PMC5303829

[B93] Stoecklin G. Mayo T. Anderson P. (2006). ARE-mRNA degradation requires the 5'-3' decay pathway. EMBO Rep. 7, 72–77. 10.1038/sj.embor.7400572 16299471 PMC1369226

[B94] Stowell J. a. W. Webster M. W. Kogel A. Wolf J. Shelley K. L. Passmore L. A. (2016). Reconstitution of targeted deadenylation by the ccr4-not complex and the YTH domain protein Mmi1. Cell Rep. 17, 1978–1989. 10.1016/j.celrep.2016.10.066 27851962 PMC5120349

[B95] Stupfler B. Birck C. Seraphin B. Mauxion F. (2016). BTG2 bridges PABPC1 RNA-binding domains and CAF1 deadenylase to control cell proliferation. Nat. Commun. 7, 10811. 10.1038/ncomms10811 26912148 PMC4773420

[B96] Takahashi A. Suzuki T. Soeda S. Takaoka S. Kobori S. Yamaguchi T. (2020). The CCR4-NOT complex maintains liver homeostasis through mRNA deadenylation. Life Sci. Alliance 3, e201900494. 10.26508/lsa.201900494 3 32238456 PMC7119370

[B97] Tan D. Zhou M. Kiledjian M. Tong L. (2014). The ROQ domain of Roquin recognizes mRNA constitutive-decay element and double-stranded RNA. Nat. Struct. Mol. Biol. 21, 679–685. 10.1038/nsmb.2857 25026078 PMC4125485

[B98] Tang T. T. L. Stowell J. Hill C. H. Passmore L. A. Hill C. H. Passmore L. A. 2019. The intrinsic structure of poly(A) RNA determines the specificity of Pan2 and Caf1 deadenylases. Nat. Struct. Mol. Biol. 26, 433–442. 10.1038/s41594-019-0227-9 31110294 PMC6555765

[B99] Ukleja M. Cuellar J. Siwaszek A. Kasprzak J. M. Czarnocki-Cieciura M. Bujnicki J. M. (2016). The architecture of the *Schizosaccharomyces pombe* CCR4-NOT complex. Nat. Commun. 7, 10433. 10.1038/ncomms10433 26804377 PMC4737751

[B100] Wahle E. Winkler G. S. (2013). RNA decay machines: Deadenylation by the Ccr4-Not and Pan2-Pan3 complexes. Biochim. Biophys. Acta 1829, 561–570. 10.1016/j.bbagrm.2013.01.003 23337855

[B101] Wang H. Morita M. Yang X. Suzuki T. Yang W. Wang J. (2010). Crystal structure of the human CNOT6L nuclease domain reveals strict poly(A) substrate specificity. EMBO J. 29, 2566–2576. 10.1038/emboj.2010.152 20628353 PMC2928688

[B110] Webster M. W. Chen Y. H. Stowell J. A. W. Alhusaini N. Sweet T. Graveley B. R. (2018). mRNA deadenylation is coupled to translation rates by the differential activities of Ccr4-not nucleases. Mol. Cell 70, 1089–1100. 10.1016/j.molcel.2018.05.033 29932902 PMC6024076

[B102] Winkler G. S. (2010). The mammalian anti-proliferative BTG/Tob protein family. J. Cell Physiol. 222, 66–72. 10.1002/jcp.21919 19746446

[B103] Wu Q. Medina S. G. Kushawah G. Devore M. L. Castellano L. A. Hand J. M. (2019). Translation affects mRNA stability in a codon-dependent manner in human cells. Elife 8, e45396. 10.7554/eLife.45396 8 31012849 PMC6529216

[B104] Xie J. Kozlov G. Gehring K. (2014). The "tale" of poly(A) binding protein: The MLLE domain and PAM2-containing proteins. Biochim. Biophys. Acta 1839, 1062–1068. 10.1016/j.bbagrm.2014.08.001 25120199

[B105] Yamashita A. Chang T. C. Yamashita Y. Zhu W. Zhong Z. Chen C. Y. (2005). Concerted action of poly(A) nucleases and decapping enzyme in mammalian mRNA turnover. Nat. Struct. Mol. Biol. 12, 1054–1063. 10.1038/nsmb1016 16284618

[B106] Yang X. Morita M. Wang H. Suzuki T. Yang W. Luo Y. (2008). Crystal structures of human BTG2 and mouse TIS21 involved in suppression of CAF1 deadenylase activity. Nucleic Acids Res. 36, 6872–6881. 10.1093/nar/gkn825 18974182 PMC2588512

[B107] Yi H. Park J. Ha M. Lim J. Chang H. Kim V. N. (2018). PABP cooperates with the CCR4-NOT complex to promote mRNA deadenylation and block precocious decay. Mol. Cell 70, 1081–1088.e5. 10.1016/j.molcel.2018.05.009 29932901

[B108] Zaessinger S. Busseau I. Simonelig M. (2006). Oskar allows nanos mRNA translation in Drosophila embryos by preventing its deadenylation by Smaug/CCR4. Development 133, 4573–4583. 10.1242/dev.02649 17050620

[B109] Zhang Q. Pavanello L. Potapov A. Bartlam M. Winkler G. S. (2022). Structure of the human Ccr4-Not nuclease module using X-ray crystallography and electron paramagnetic resonance spectroscopy distance measurements. Protein Sci. 31, 758–764. 10.1002/pro.4262 34923703 PMC8862426

